# A descriptive survey of substance use treatment facilities in Uasin Gishu County Kenya

**DOI:** 10.1186/s12913-022-08051-w

**Published:** 2022-05-14

**Authors:** Florence Jaguga, Matthew Turissini, Julius Barasa, Mercy Kimaiyo, Joash Araka, Lily Okeyo, Edith Kwobah

**Affiliations:** 1 Moi Teaching & Referral Hospital, P.O. BOX 3-30100, Eldoret, Kenya; 2 Department of Internal Medicine, Indiana University, Bloomington, USA; 3 Academic Model Providing Access to Healthcare, PO BOX 4606, Eldoret, Kenya

**Keywords:** Substance use, Treatment, Facilities, Kenya

## Abstract

**Background:**

Substance use disorders are a major problem in Uasin Gishu County, Kenya. The objective of this study was to describe the existing resources within substance use treatment facilities in the County, with the aim of guiding policy and interventions.

**Methods:**

This was a cross-sectional study. We collected data from six substance use treatment facilities within Uasin Gishu County between August and November 2021. We used a researcher-designed questionnaire to collect information on: availability of in-patient and out-patient services; facility ownership (private-for-profit vs government-run); bed capacity; mode of payment for services; cost of services; availability of medicines for substance use treatment; and staffing characteristics. Descriptive statistics were used to summarize the data.

**Results:**

One facility was run by the National government and the rest were private-for-profit. Uasin Gishu County government had no substance use treatment facility of its own. The total number of beds available within the six facilities was 174 against a population of 1.1 million. All six facilities had stocked at least one medication for substance use disorder treatment. None of the facilities had buprenorphine, buprenorphine naloxone, or methadone. Out-of pocket was the most common mode of payment for services with patients paying using this mode in all the six facilities. Only one facility was accredited by the National Hospital Insurance Fund (NHIF). All facilities had at least one certified addiction counselor and at least one psychologist. Half of the facilities did not have a nurse and two did not have a doctor. The qualification held by most staff was addiction counseling with 41.3% of them having achieved this qualification.

**Conclusion:**

The facilities were well staffed with psychologists and addiction counselors. Gaps were found as regards bed capacity, use of pharmacotherapy, insurance coverage and availability of nursing staff and doctors. We recommend that the County government in collaboration with key stakeholders invests in substance use treatment in order to address the high burden of substance use disorders in Uasin Gishu County.

## Introduction

Substance use is a major public health problem globally. In the 2017 Global Burden of Disease study, substance use disorders (SUDs) were the second leading cause of disability among the mental disorders with 31,052,000 (25%) Years Lived with Disability (YLD) attributed to them [1]. In 2016, harmful alcohol use resulted in 3 million deaths (5.3% of all deaths) worldwide and 132.6 (5.1%) million disability-adjusted life years (DALYs) [2]. Tobacco use, the leading cause of preventable death, kills more than 8 million people annually [3]. Further compounding this situation, is the fact that SUDs are associated with social costs as high as I$ 800 per head [4], emanating from their impact on productivity, crime and health systems [5].

In Kenya, over 10% of persons aged between 15 and 65 years have an alcohol use disorder, with most of them (60%) having the severe form [6]. In fact, the country has one of the highest total DALYs (54,000) from alcohol use disorders in Africa [7]. The prevalence rates for tobacco, khat and cannabis use disorders are 6.8, 3.1 and 0.8% respectively [6]. A recent key population size estimate reported that the number of Persons Who Inject Drugs (PWIDs) in Kenya was 35,784 [8].

Uasin Gishu County is located in the western part of Kenya, and is home to about 1.1 million people [9]. The prevalence rates of substance use among residents of the County are high. In 2020, there were 906 PWIDs within Uasin Gishu County [8]. A study conducted among inmates in Eldoret town, the administrative Capital for the County, reported the prevalence rate of any substance use to be 66.1% [10]. Children and youth within the County have not been spared. A study conducted among university students within the region reported that 69.8% had ever used at least one substance in their lifetime [11]. In 2019, a nationwide survey conducted among primary school children (grade 5–8) across all 47 counties in Kenya, reported that pupils from Uasin Gishu County had the highest rates of lifetime tobacco use (20.0%) and the second highest rates of lifetime alcohol use (17.5%) countrywide [12] Among street connected youth in the County, the lifetime prevalence rate of any substance use was reported as 74% by Embleton et al. [13].

Despite this, the County Government has not prioritized programs and interventions that address substance use. In the 2018–2022 County Integrated Development Plan, the high burden of substance use among the youth is acknowledged yet no treatment and prevention programs have been planned to address the problem [14]. In line with the Constitution of Kenya 2010 [15], health service delivery is a devolved function and is the responsibility of the County governments.

The Academic Model Providing Access to Healthcare (AMPATH) is a partnership between Moi University, Moi Teaching and Referral Hospital in Kenya, Indiana University in North America, and the Kenyan Government [16]. The original mandate of AMPATH was to improve care and promote research for HIV in western Kenya (including Uasin Gishu County). AMPATH has since extended its mandate to chronic disease management including mental health. In 2020, the AMPATH mental health program received a grant to improve the quality of mental health and substance use services within Uasin Gishu County. In order to plan for interventions, the AMPATH mental health program undertook a baseline survey to map the existing mental health and SUD treatment services within Uasin Gishu County. This paper describes the substance use treatment services. The mental health services will be reported in a separate paper.

Treatment for SUDs in Kenya is mainly delivered through residential or in-patient facilities accredited by the National Authority for the Campaign Against Alcohol and Drug Abuse (NACADA) [17]. The treatment program typically runs over a 90-day period. As at August 2021, Uasin Gishu County had six accredited SUD treatment facilities. Out of these, only one is government-run and is under the management of the National government. The rest are privately-owned [18]. The SUD treatment facilities are mostly located within urban and peri-urban regions of the County. Five of the facilities are located within a 10 km radius of Eldoret town (the administrative capital of Uasin Gishu County), and one is located 20 km from Eldoret town. While literature indicates that other forms of substance use programs exist within Uasin Gishu County [19], there is no comprehensive directory listing of substance use services offered other those listed in the NACADA website [17].

This study therefore sought to describe the resources available within substance use treatment facilities located in Uasin Gishu County, and identify gaps in order to guide interventions by the AMPATH mental health program and other key stakeholders. This work aligns with target 3.5 of the sustainable development goals [20] and the Kenya mental health action plan 2021–2025 [21] both of which require that treatment and prevention for SUDs is strengthened.

## Methods

### Study design

We collected this data as part of a larger cross-sectional survey whose aim was to map the mental health services in western Kenya. Data presented in this paper was collected from six substance use treatment facilities in Uasin Gishu County, between August and November 2021.

### Recruitment

We sent an invitation to participate in the survey and a consent form to the respective substance use treatment facility heads via email. Interview dates and times were agreed upon via email or during follow-up phone calls for those who failed to respond to the emails. The substance use treatment facilities contacted were those accredited by NACADA at the time of the survey [18].

### Data collection

In the absence of a standardized tool for collecting data on substance use treatment services, the investigators prepared a questionnaire seeking to obtain information on:

- Availability of in-patient and out-patient services
- Facility ownership: Private-for-profit vs government run
- Number of beds within the facilities including bed allocation to females, males, children and adolescents
- Mode of payment for services; cost of services
- Availability of medicines for substance use treatment based on those listed as essential in the Kenya Essential Medicines List 2019 [22]
- Staffing characteristics: the number of health care providers (HCPs), age, gender, mental health or health qualifications. Because a number of staff had more than one health or mental health-related qualifications, we recorded the highest level of mental health qualification or highest level of health qualification where the staff had no mental health training. In addition we obtained information on whether the staff had received specific addiction certification training. In Kenya the addiction certification is received through training based on the universal treatment curriculum (UTC). The training is regularly administered by two organizations i.e. NACADA or the Support for Addictions Prevention and Treatment in Africa (SAPTA) [23].

We collected data from the facility heads during face-to- face interviews. Written informed consent was obtained from all facility representatives before data collection.

### Data analysis

Data were entered into an excel data base and checked for completeness and accuracy. Facility heads were re-contacted via phone call in case clarifications were needed or there was incomplete data. Data were summarized using descriptive statistics. Continuous data were summarized using means, and categorical data using frequencies and percentages.

## Results

### General characteristics of the substance use treatment facilities

All six facilities participated in the survey. Out of these, one facility was run by the National government, and was located within a general health facility. There was no County government run SUD treatment facility. The rest were stand-alone facilities specializing in SUD treatment, and were private-for-profit. All facilities provided in-patient substance use treatment while only two additionally provided out-patient care.

### Bed capacity

The total number of beds available within the six facilities was 174 with an average of 29 beds per facility (range 16–60 beds). Of these, 33 (19.0%) had been allocated to females while the rest were allocated to males. None of the facilities had dedicated beds for children and adolescents. Only 16 beds (9.2%) were available within the government run facility (Table 1).

**Table 1:** Bed capacity within the substance use treatment facilities (*n* = 174)

	Government-run n(%)	Privately for profit n(%)	Total
Male	11 (6.3%)	130 (74.7%)	141 (81.0%)
Female	5 (2.9%)	28 (16.1%)	33 (19.0%)
Children and adolescents	0 (0%)	0 (0%)	0 (0%)
Total	16 (9.2%)	158 (90.8%)	174 (100%)

### Availability of medicines for SUD treatment

Nicotine replacement therapy was available in four facilities. Naltrexone and bupropion were stocked and dispensed in the government-run facility only. None of the facilities had buprenorphine, buprenorphine-naloxone or methadone (Table 2).

**Table 2:** Medication availability within the SUD treatment facilities (*n* = 6)

Medication	Government	Private for profit	Total^**a**^ n(%)
Nicotine replacement therapy	1	3	4 (66.7%)
Naltrexone	1	0	1 (16.7%)
Bupropion	1	0	1 (16.7%)
Buprenorphine	0	0	0 (0%)
Buprenorphine-naloxone	0	0	0 (0%)
Methadone	0	0	0 (0%)

^**a**^figures do not add up to 100% because some facilities had more than one medication

### Payment for services and cost of treatment

Out-of pocket was the most common mode of payment for services with patients paying using this mode in all the six facilities. Only one facility was accredited by the government managed National Hospital Insurance fund (NHIF), while one allowed payment using private insurance providers (Table 3). The cost of a 90-day in-patient program ranged from US$ 700 to 2000.

**Table 3:** Mode of payment for SUD treatment services (*n* = 6)

	Government-run	Private for profit	Total^**a**^ n(%)
NHIF	1	0	1 (16.7%)
Private insurance providers	0	1	1 (16.7%)
Out of pocket	1	5	6 (100%)

^a^figures do not add up to 100% because some facilities had more than one mode of payment

### General characteristics HCPs working within the facilities

There were a total of 63 HCPs working within the six facilities. Of these, 57 (90.5%) were working full-time, while 6 (9.5%) were available on call basis. Most of the staff 37 (58.7%) were female. The mean age of the staff was 39.7 years (Table 4).

**Table 4:** General characteristics HCPs working within the facilities (*n* = 63)

Staff characteristics	Mean/ n (%)
**Demographics:**	
Mean age	39.7 years
Female	37 (58.7%)
**Working characteristics:**	
Full time HCPs	57 (90.5%)
On-call	6 (9.5%)
**Sector:**	
Private	45 (71.4%)
Government	18 (28.6%)

### Staff availability within each facility

All facilities had at least one certified addiction counselor and at least one psychologist. Four facilities had at least one resident psychiatrist, or one who was available on-call. Half of the facilities had at least one nurse (Table 5).

**Table 5:** Number of facilities with at least one staff of each HCP cadre (*n* = 6)

Staff qualifications	Government	Private for profit	Total n(%)
Psychologists	1	5	6 (100.0%)
Addiction counselors	1	5	6 (100.0%)
Nurses	1	2	3 (50.0%)
Psychiatrists (full-time and on-call)	1	3	4 (66.7%)
Other doctors	1^a^	3	4 (66.7%)
Social workers	1	3	4 (66.7%)
Occupational therapists	1	1	2 (33.3%)
Nutritionists	1	2	3 (50.0%)
Physiotherapists	1^a^	1	2 (33.3%)
Community health workers	0	1	1 (16.7%)

^a^Physiotherapists and other doctors were not fully stationed at the substance use unit within the government based facility but were available for consultation from the other hospital departments when needed

### Characteristics of HCPs by qualifications

The qualification held by most staff was addiction counseling with 41.3% of them having achieved this qualification. This was followed by general nursing (20.6%) and psychiatry (14.3%). Because psychotherapy is core to treatment for SUDs, we segregated the psychologists according to level of qualification. Out of the 12 HCPs with training in psychology, 7 (58.3%) had a qualification of a degree or higher. Eight out of the nine HCPs who were available on-call were doctors (Table 6).

**Table 6:** Characteristics of HCPs by qualifications (*n* = 63)

Staff cadre and qualifications	Government	Private	Total	Percentages^**a**^
**Psychologists:**				
Masters in Clinical psychology	1	1	2	3.2%
Degree in medical psychology	0	2	2	3.2%
Degree in counseling psychology	1	2	3	4.8%
Diploma in counseling psychology	0	3	3	4.8%
Certificate in counseling psychology	0	1	1	1.6%
**Addiction certification training:**				
UTC training	12	13	25	39.7%
**Nurses:**				
General nursing	8	5	13	20.6%
Psychiatric nursing	0	1	1	1.6%
**Doctors:**				
Psychiatrists working fulltime	4	1	5	7.9%
Psychiatrists on-call	0	4	4	6.3%
Physician (internal medicine) on-call	0^b^	1	1	1.6%
General doctor on call	0	3	3	4.8%
**Other:**				
Social workers	1	6	7	11.1%
Occupational therapists	2	1	3	4.8%
Nutritionists (full-time)	1	0	1	1.6%
Nutritionist on call	0	1	1	1.6%
Physiotherapists	0^b^	1	1	1.6%
Community health workers	0	1	1	1.6%
Lay providers/peers^c^	0	5	5	7.9%

^a^Percentages add up to a figure greater than 100 because some staff had addiction counseling in addition to a health or mental health qualification

^b^Within the government facility, these staff cadres were not fully stationed at the substance use treatment unit but were available for consultation if need be. Counting them as substance use staff would have over-inflated the SUD workforce within Uasin Gishu County

^c^Peer providers are those who offered counseling or other support to the patients but had no training in health, mental health, or addiction counseling

## Discussion

This is one of the few studies conducted in Kenya with the aim of describing the characteristics of substance use treatment facilities in Kenya. A study conducted by Ndege and Njenga [17] in 2009 across Kenya, described substance use facility ownership, staffing qualifications and services offered. Our current study extends this work by assessing bed capacity, medication availability, cost of services, and mode of payment for services.

### Bed capacity

The total number of beds in the six facilities was 174 resulting in 16 beds per 100,000 population.. Of the 174 beds, 16 were available within a government-run facility, and the rest were found within specialized standalone private SUD treatment facilities. We did not find international or local data on the minimum and optimal number of beds per population for SUD treatment. Nonetheless, the bed capacity within Uasin Gishu County is likely inadequate for its population, given the high rates of substance use and misuse in the County [11, 12]. Moreover, the neighboring counties of Kakamega, Trans-Nzoia, Baringo and Elgeyo Marakwet with a total population of about 4 million have no substance use treatment facilities [18], and patients from these counties often seek care from Uasin Gishu County. The limited number of beds coupled with the fact that most of the beds (90.8%) were available within private-for-profit facilities whose services are often costly, highlights the limited access to treatment for SUDs within Uasin Gishu County. It is also an indication that there is little investment in SUD treatment by the County government despite the high documented burden. As the main stakeholders in the management of SUDs, the County Department of Health, as well as NACADA ought to work to increase the capacity for SUD treatment within the County. As a start, beds could be allocated within existing health facilities and HCPs assigned to manage patients after brief trainings like the UTC certification. This strategy is in line with the Kenya Mental Health Action Plan 2021–2025, which calls for strengthening of SUD treatment services within existing health and mental health services [21].

Only, 33 (19.0%) of the beds had been allocated to females and no facility had beds dedicated for children and adolescents. Previously, SUDs have been associated with being male. With the evolving of gender roles, the gap in substance use between males and females is closing and more beds need to be allocated to females. Recent surveys show significant rates of substance misuse among children and adolescents [12] and beds need to be dedicated for this vulnerable population as well.

Limited bed capacity for SUD treatment has also been noted elsewhere. In Nigeria, a survey conducted in 2011 reported that the entire country had a total of 566 beds for a population of 154 million resulting in a bed to population ratio of 1:272000 [24]. In 2020, there were 71,433 beds available for SUD treatment across the US, against a population of 329.5 million that year resulting in a bed to population ratio of 1:4600 [25].

### Availability of medicines for substance use treatment

Overall, there was limited use of pharmacotherapy in the management of SUDs within the County. Nicotine replacement therapy was available in four facilities; naltrexone and bupropion in one facility; and none of the facilities had buprenorphine, buprenorphine-naloxone or methadone. This finding could be related to a number of reasons. First, it is likely that many HCPs have limited knowledge on the use of medication-assisted therapy in substance use treatment. Secondly, the medication is not easily available because some of them are strictly regulated, and also due to the fact that their costs are prohibitive. For example a 50 mg tablet of naltrexone costs about $4. This would translate to a total cost of $120 for a month’s dose. Thirdly, limited use of medication for substance use could be related to the fact that half of the facilities did not have nursing staff.

Medication for opioid use disorder was not available in any of the facilities. This is concerning given that there has been a rise in heroin use as well as prescription opioid use in the western part of the country over the past decade. Between 2007 and 2017, the prevalence rates of lifetime heroin use for persons aged 15–65 years increased from 0.2 to 0.5% in Rift Valley, from 0 to 0.4% in Western province [6]. Even though the prevalence of opioid use disorder is low globally and in Kenya, the mortality and morbidity associated with opioid use disorders is typically high. In 2015, 76% of all deaths from SUDs were as a result of opioid use disorder [7]. The lack of medication for opioid use disorder could be related to a number of reasons. The cost of buprenorphine is high, about US$100 for a month’s dose. Because of their abuse potential, both drugs are heavily regulated. Facilities are required to meet minimum regulatory, staffing, and infrastructure requirements prior to being licensed to stock and dispense these drugs. In the US, only 10% of SUD treatment facilities offered methadone treatment in 2020 [25].

In order to address the limited medication use within SUD treatment facilities within Uasin Gishu County, staff should receive education on the use of pharmacotherapy for the treatment of SUDs. This can be done through continuous professional development sessions organized by key stakeholders. NACADA requires that residential/in-patient facilities have adequate facilities and skilled staffing that allow for proper storage, prescription and administration of pharmacotherapy. We found that a half of the facilities did not have nursing staff and two did not have a doctor either working full-time or on-call. This may be a hindrance to pharmacotherapy use. Facilities ought to ensure that they have registered medical practitioners and nursing staff so that patients can benefit from evidence based pharmacotherapies. Finally, adequate insurance coverage for SUD treatment will ensure that the high cost of medication does not constitute a barrier to accessing treatment.

### Mode of payment for substance use treatment services

Out-of pocket was the most common mode of payment with patients paying using this method in all six facilities. This is consistent with findings in a US survey which reported that the most commonly accepted payment type at substance use treatment facilities was cash or self-payment (90%). Only one facility in Uasin Gishu County was NHIF accredited, while only one allowed for payment using other private insurance companies. It is important to note that NHIF only covers a fraction (55%) of the SUD treatment costs in government substance use treatment facilities. In the US, 74 and 71% of substance use treatment facilities allow for payment using private and public health insurance respectively [25].

With treatment costs ranging from US$700–2000, paying out-of pocket for substance use treatment is out of reach for Uasin Gishu County residents and can be impoverishing. The average household income in Kenya is US$100 per month.

The high treatment costs and limited insurance coverage have implications for access to treatment. Many residents of Uasin Gishu County who need SUD treatment are unable to afford it, therefore go untreated. The County therefore continues to suffer the heavy health and socio-economic impacts of untreated substance use.

The mental health taskforce report recommends that NHIF provides comprehensive coverage for outpatient and inpatient mental health and SUD treatment, and directs that the Insurance Regulatory Authority ensures that there is no discrimination as regards coverage for mental health conditions including SUDs [26]. These should be implemented. Key stakeholders should prioritize efforts to advocate for better coverage for SUDs by insurance providers.

To further address the financial barriers to SUD treatment, less costly and more accessible means of treatment for SUDs should be explored. Treatment for low to moderate risk SUDs can be delivered in community settings and using task-shifted strategies. These have been piloted already on a small scale within Uasin Gishu County with some success and could be adopted and scaled up by the County government [19, 27]. Another cost-effective option would be to integrate substance use screening and brief intervention into primary health care [28]. This has been successfully implemented in other low-to-middle-income settings [29]. For those with severe SUDs, the duration of in-patient stay could be shortened from the current 90-day program that is routinely practiced in Kenya, to a 6-week program to make it more affordable [30]. Intensive out-patient treatment options could also be explored.

### Staffing characteristics

Overall, the facilities were well staffed with core specialist mental health and substance use service providers. All facilities had at least one certified addiction counselor and at least one psychologist, while four out of six facilities had a psychiatrist providing services either full-time or part time. This highlights the importance that the facilities have assigned to providing specialized and evidence based approaches in the management of SUDs.

Three out of four of the staff available on-call basis were doctors. This is not surprising given the high doctor to population ratios in Kenya. It is commendable that five out of six facilities either had a full-time doctor or one that was available on-call. SUDs are often associated with comorbid physical and mental disorders. Having a doctor available to review ensures that these disorders are identified and addressed in a timely and comprehensive manner. Only three out of six facilities however, had nurses casting doubt on the quality of care as regards medication administration, and patient monitoring in the facilities without this cadre.

We acknowledge a number of limitations. First, we did not use a structured tool to collect this data. The World Health Organization has made attempts at developing a standardized tool for mapping substance use services but this is yet to be validated [31]. Secondly, we may have missed out other potential substance use treatment services e.g. non-governmental organizations and support groups because of lack of a comprehensive database. Nonetheless our study provides important information on the resources available within SUD treatment facilities within a high burden County in Kenya.

## Conclusion

In sum, substance use treatment services within Uasin Gishu County were largely offered by the private sector. There was a shortage of beds for substance use treatment. Services were costly and were poorly covered by insurance. There was limited use of medication assisted therapy in the management of SUDs. Specialist mental health staffs were available in most of the facilities indicating recognition of SUDs as a mental health problem. Some facilities did not have nursing staff and medical practitioners as required by NACADA. We recommend that the AMPATH mental health program in collaboration with the County government, NACADA, and other stakeholders, work to address these gaps.

## Data Availability

The datasets used and/or analyzed during the current study are available from the corresponding author on reasonable request.

## Abbreviations

AMPATH: Academic Model Providing Access to Healthcare
DALY: Disability Adjusted Life Year
HCP: Healthcare provider
HIV: Human Immunodeficiency Virus
I$: International Dollar
KEML: Kenya Essential Medical List
KNBS: Kenya National Bureau of Statistics
NACADA: National Authority for the Campaign Against Alcohol and drug Abuse
NHIF: National Hospital Insurance Fund
PWID: Persons Who Inject Drugs
SAPTA: Support for Addictions Prevention and Treatment in Africa
SUD: Substance Use Disorder
UTC: Universal Treatment Curriculum
YLD: Years Lived with Disability
